# The Role of Lung Volume, Age, and Body Mass Index in Determining Obstructive Sleep Apnea Severity

**DOI:** 10.3390/medicina62061197

**Published:** 2026-06-22

**Authors:** Enes Gul, Ömer Tamer Doğan, Neslihan Taş, Irfan Atik, Ismail Salk

**Affiliations:** 1 Department of Radiology, Faculty of Medicine, Sivas Cumhuriyet University, 58140 Sivas, Turkey; irfanatik_91@hotmail.com (I.A.); ismailsalk@gmail.com (I.S.); 2 Department of Pulmonary Medicine, Faculty of Medicine, Sivas Cumhuriyet University, 58140 Sivas, Turkey; tdogangs@gmail.com (Ö.T.D.); drneslihan.tas@gmail.com (N.T.)

**Keywords:** obstructive sleep apnea syndrome (OSAS), Apnea–Hypopnea Index (AHI), lung volume, spirometry, computed tomography (CT)

## Abstract

*Background and Objectives:* It is well established that obstructive sleep apnea syndrome (OSAS) is associated with functional lung volumes. The aim of this study was to investigate the relationship between morphological lung volume and the severity of OSAS. *Materials and Methods:* Adult patients evaluated for sleep disorders between January 2020 and January 2024 were retrospectively reviewed. Patients with an AHI greater than 5, who underwent both spirometry and thoracic CT within a three-month interval, were included. Spirometric functional volume and morphological CT lung volume were assessed. Associations between OSAS severity and both functional and morphological lung volumes were analyzed. *Results:* A total of 195 patients were enrolled, of whom 166 had CT scans suitable for lung volume assessment. Among all patients, 20 (10.3%) were in the mild, 39 (20.0%) in the moderate, and 136 (69.7%) in the severe OSAS group. Ordinal regression analysis was performed to evaluate the factors influencing these categories. Age (*p* < 0.001) and BMI (*p* < 0.001) were positively correlated with disease severity, whereas female sex was associated with a lower risk of severe disease (*p* = 0.003). *Conclusions:* Functional and morphological lung volumes did not affect OSAS severity. Functional and morphological lung volumes were positively correlated with each other. Both morphological and functional lung volumes showed negative correlations with BMI.

## 1. Introduction

Sleep apnea syndrome encompasses two distinct subtypes: obstructive sleep apnea syndrome (OSAS) and central sleep apnea syndrome (CSAS). These entities differ fundamentally in their pathophysiology; OSAS arises from mechanical upper airway obstruction, whereas CSAS results from impaired central respiratory drive. The present study focused exclusively on patients with OSAS. Obstructive sleep apnea syndrome (OSAS) is characterized by partial airflow limitation (hypopnea) and complete cessations (apnea) of ventilation. The gold standard for diagnosing OSAS is polysomnography (PSG). The severity of OSAS can be classified according to the Apnea–Hypopnea Index (AHI) [1,2]. Its prevalence increases in parallel with obesity. A systematic review investigating the prevalence of OSAS in the general population reported prevalence ranging from 9% to 38% when AHI ≥ 5 was used as the threshold, and from 6% to 17% when AHI ≥ 15 was used [3].

A study in children demonstrated that forced vital capacity (FVC) (% predicted) is reduced in those with OSAS [4]. Moreover, individuals at high risk of OSAS, as determined by a multivariable apnea prediction index, exhibited a faster decline in forced vital capacity (FVC) and forced expiratory volume in one second (FEV_1_) [5]. In patients with overlap syndrome, a positive correlation has been reported between FEV_1_ and AHI [6]. These findings suggest an association between OSAS and pulmonary function.

To our knowledge, a few studies have examined the relationship between OSAS severity and pulmonary function. Furthermore, no study has specifically investigated the association between tomographic lung volume and OSAS in this patient population. Based on the available literature, our study appears to be the first to address this question. Therefore, the aim of this study was to explore the relationship between OSAS severity, functional lung volumes (measured by spirometry), and tomographic lung volume.

## 2. Materials and Methods

This study was designed as a retrospective, single-centre, cross-sectional analysis. The ethics committee decision was made by the Sivas Cumhuriyet University Health Sciences Research Ethics Committee (decision number 2024-11/24, dated 21 November 2024). After obtaining ethical approval, patients who underwent polysomnography between January 2020 and January 2024 were retrospectively reviewed.

Inclusion criteria:

- Age ≥ 18;
- AHI > 5;
- Availability of both spirometry and thoracic computed tomography (CT);
- Interval between spirometry and thoracic CT is less than three months.

Exclusion criteria:

- Diagnosis of central sleep apnea syndrome (CSAS);
- Inability to perform an acceptable spirometry test;
- Spirometry results consistent with chronic obstructive pulmonary disease (COPD) or asthma;
- Total sleep time < 4 h during polysomnography;
- Presence of known chronic lung diseases (e.g., occupational lung disease, pulmonary fibrosis, tuberculosis, bronchiectasis, etc.);
- Patients receiving non-invasive ventilation.

**Spirometry**

Routine pulmonary function test results, performed prior to polysomnography, were evaluated in patients diagnosed with OSAS. Spirometric assessment was conducted in accordance with the 2023 guidelines of the American Thoracic Society (ATS) and the European Respiratory Society (ERS). Reference values were calculated using Global Lung Initiative (GLI) 2012 equations, which account for age, height, sex, and ethnicity. Key parameters recorded included Forced Vital Capacity (FVC), Forced Expiratory Volume in one second (FEV_1_), and the FEV_1_/FVC ratio.

**Polysomnography**

Physiological parameters during sleep were assessed overnight using full-night PSG. All recordings were performed in an accredited sleep laboratory according to the standards of the American Academy of Sleep Medicine (AASM).

The following parameters were recorded:

- EEG (electroencephalogram): for assessment of brain waves;
- EOG (electrooculogram): for monitoring eye movements;
- EMG (electromyogram): for evaluating chin and leg muscle activity;
- ECG (electrocardiogram): for cardiac rhythm monitoring;
- Respiratory parameters: nasal airflow (via nasal cannula), thoracic and abdominal movements (via belts);
- Oxygen saturation: measured with pulse oximetry;
- Body position and snoring sounds were also recorded.

Polysomnography was performed using Embla^®^ S4000 and Embla^®^ N 7000 devices (Embla Systems, Thornton, CO, USA). The results were scored by two experienced sleep technicians according to standard criteria [7]. Patients diagnosed with OSAS were classified into three categories based on the number of respiratory events per hour: mild OSAS (AHI 5–14.9/h), moderate OSAS (AHI 15–29.9/h), and severe OSAS (AHI > 30/h). An AHI < 5 was considered normal [1].

**Computed Tomography**

Chest CT scans were performed using a 128 detector scanner (GE Revolution EVO, MIL, Seoul, Republic of Korea). Images were obtained from the lung apices to the bases. Scan parameters were as follows: slice thickness, 0.625 mm; 100 kV; auto mA (range, 80–250); and a large field of view. The images were taken in the supine position, during maximal inspiration, and without contrast.

**Image analysis**

**Figure 1:**
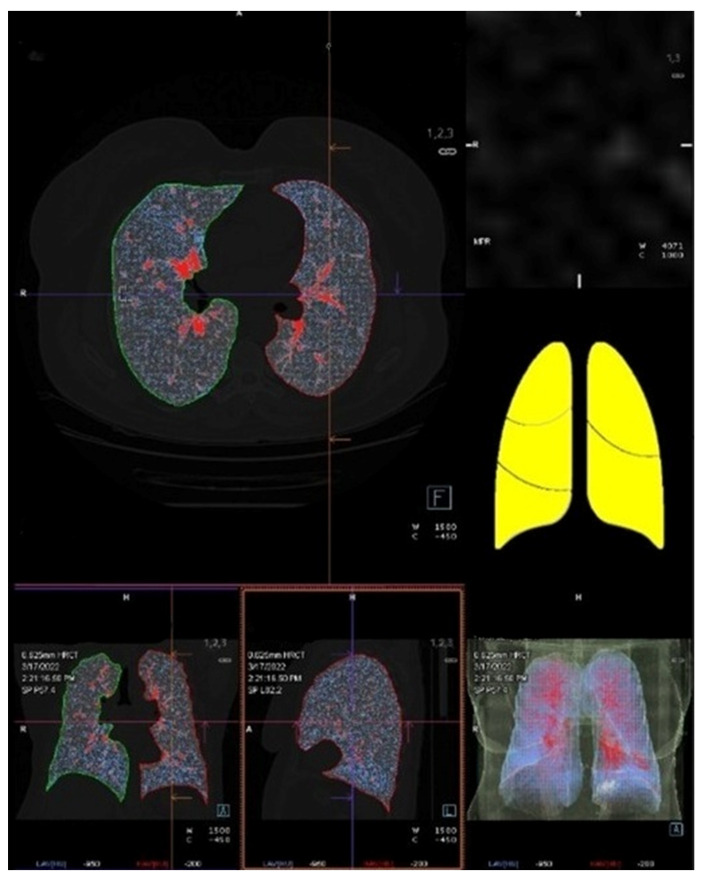
Tomographic lung volume measurement with a fully automated method using Syngo via software.

All thoracic CT scans were independently reviewed by two experienced thoracic radiologists specialised in cross-sectional imaging to identify and exclude pathological findings. After consensus, cases without lung pathology (infection/consolidation, significant atelectasis, pleural effusion, significant emphysema, interstitial lung disease, etc.) were included. The raw images were uploaded to Siemens Syngo.via software version VB60S_HF01, and total lung volume was calculated automatically (Figure 1).

**Statistical analysis**

All statistical analyses were performed using SPSS version 23.0 (IBM Corp., Armonk, NY, USA). Descriptive statistics were reported as mean ± standard deviation, median, minimum, and maximum values for continuous variables, and as counts with percentages for categorical variables. The normality of data distribution was assessed using skewness and kurtosis.

Group comparisons were conducted using one-way analysis of variance (ANOVA) for continuous variables and the Mann–Whitney U test for non-normally distributed data. Post hoc analyses were performed where applicable. Associations between continuous variables were evaluated using both Pearson and Spearman correlation coefficients.

Ordinal logistic regression analysis was employed to determine the independent predictors of OSAS severity, classified into mild, moderate, and severe categories. The complementary log-log link function was selected for this model due to the markedly asymmetric distribution of OSAS severity categories in our cohort, with a disproportionately high proportion of severe cases (69.7%). This link function is particularly appropriate for right-skewed ordinal outcomes in which higher categories are more probable. A generalized linear model (GLM) was additionally conducted with the Apnea–Hypopnea Index (AHI) as the dependent variable to identify significant predictors.

Receiver operating characteristic (ROC) curve analyses were performed to assess the discriminative ability of age, BMI, and smoking history (pack-years) for severe OSAS, with optimal cut-off values determined using the Youden index.

A two-tailed *p*-value < 0.05 was considered statistically significant for all analyses.

## 3. Results

After applying the exclusion criteria, 195 patients were included in the study. The mean age was 53.3 ± 11.7 years, ranging from 21 to 81 years. CT scans of 166 patients were suitable for lung volume assessment. Among the 195 patients, 48 were active smokers (14 women and 34 men). The descriptive characteristics of the study population are presented in Table 1.

Of the patients, 76 were female and 119 were male. Descriptive statistics of men and women are shown in Table 2.

**Figure 2:**
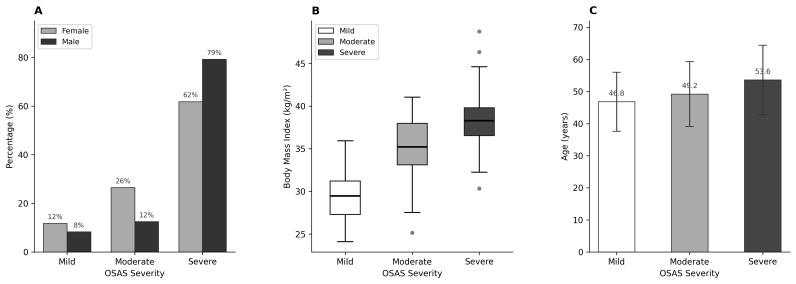
The relationship between OSAS severity and sex (**A**), body mass index (BMI) (**B**) and age (**C**).

Among the study population, 20 patients (10.3%) were classified as mild, 39 (20%) as moderate, and 136 (69.7%) as severe. Ordinal regression analysis was conducted to evaluate the impact of parameters on these categories. Due to the clustering observed in the upper categories of the dependent variable (Severe OSAS, 69.9%), the ‘Complementary Log-log’ link function was preferred in the analysis. Model-fitting analytics indicated that the developed predictive model was statistically significant (*p* < 0.001) and demonstrated a robust fit to the data (Pearson Goodness-of-Fit, *p* = 0.221). The Nagelkerke R^2^ was 0.288, indicating that the independent variables explained 28.8% of the variance in OSAS severity. Furthermore, the assumption of proportional odds was satisfied, as confirmed by the test of parallel lines (*p* = 0.428). According to the regression model results, age (β = 0.071; *p* < 0.001) and BMI (β = 0.103; *p* < 0.001) were strongly and positively associated with increased OSAS severity. Analysis of the sex variable revealed that being female was associated with lower OSAS severity than being male (β = −1.226; *p* = 0.003). Conversely, smoking status, FVC (L), and total lung volume did not make a statistically significant independent contribution to predicting OSAS severity in this multivariate model (Table 3, Figure 2).

A General Linear Model (GLM) was conducted to identify the factors influencing OSAS severity (raw AHI values). The developed model was statistically significant (F(7, 158) = 9.665, *p* < 0.001) and accounted for 30% of the variance in AHI (R^2^ = 0.300). Levene’s test confirmed the homogeneity of error variances (*p* = 0.948). In the multivariate model, body mass index (BMI) was identified as the strongest independent predictor of AHI (F = 28.029, *p* < 0.001, ηp^2^ = 0.151). Sex emerged as the second most influential factor (F = 20.433, *p* < 0.001, ηp^2^ = 0.115), with adjusted mean AHI values significantly higher in males (56.51 ± 2.83) than in females (30.61 ± 4.44). Age also demonstrated a statistically significant effect within the model (*p* = 0.005). Conversely, CT-based Total Volume (*p* = 0.821) and FVC (L) (*p* = 0.391), along with smoking status (*p* = 0.787), showed no independent effect on AHI when BMI and other clinical factors were controlled (Table 4).

One-way ANOVA was used for group comparisons. Significant differences were observed in age and BMI (*p* < 0.001 and *p* = 0.009, respectively), but not in other parameters. Post hoc analysis revealed significant differences in age between Group 1 and Group 3 (*p* < 0.001) and between Group 2 and Group 3 (*p* = 0.001). For BMI, a significant difference was detected between group 1 and group 3 (*p* = 0.007).

These findings indicated that neither functional nor morphological lung volumes affected OSAS severity. Therefore, mild OSAS was considered the control group, while moderate and severe OSAS were combined as the patient group. Comparison using Student’s *t*-test showed significantly higher age and BMI values in the patient group (*p* = 0.001 and *p* = 0.003, respectively), while lung volumes did not differ significantly.

Correlation analysis showed a weak positive correlation between age (Pearson r = 0.29, *p* < 0.001; Spearman ρ = 0.34, *p* < 0.001), BMI (Pearson r = 0.28, *p* < 0.001; Spearman ρ = 0.29, *p* < 0.001), and AHI score. Conversely, a negative correlation was observed between FVC (% predicted) and AHI score (Pearson r = −0.18, *p* = 0.009; Spearman ρ = −0.16, *p* = 0.018).

Total morphological lung volume was strongly and positively correlated with FEV_1_ (Pearson r = 0.701, *p* < 0.001; Spearman ρ = 0.727, *p* < 0.001, n = 166) and FVC (Pearson r = 0.646, *p* < 0.001; Spearman ρ = 0.680, *p* < 0.001, n = 166).

BMI was significantly and negatively correlated with both functional and morphological lung volumes. Specifically, BMI was negatively correlated with FEV_1_ and FVC (Pearson r = −0.38 and −0.40, respectively; *p* < 0.001 for both), as well as with morphologic lung volumes (total lung volume r = −0.32, right lobe r = −0.30, left lobe r = −0.34; all *p* < 0.001). Spearman correlations confirmed these findings (total lung volume ρ = −0.30, R lobe volume ρ = −0.30, L lobe volume ρ = −0.31; all *p* < 0.001).

A partial correlation analysis was conducted to evaluate the potential confounding effect of body mass index (BMI) on the relationship between lung volume and OSAS severity. In the zero-order correlation (without controlling for any variables), a statistically significant, weak negative correlation was observed between AHI and FVC (% predicted) (r = −0.187, *p* = 0.009). However, when BMI was included in the analysis as a control variable, the correlation between lung volume and AHI diminished to the threshold of statistical significance (r = −0.140, *p* = 0.051). This finding suggests that although a substantial portion of the relationship between reduced lung volume and OSAS severity is explained by increased BMI, a potential independent mechanical effect of lung volume (*p* = 0.051) cannot be entirely ruled out.

Regarding sex differences, 10 women (13.2%) were in the mild group, 22 (28.9%) in the moderate group, and 44 (57.9%) in the severe group. Among men, 10 (8.4%) were mild, 17 (14.3%) moderate, and 92 (77.3%) severe. The Mann–Whitney U test revealed a significant difference in AHI-based OSAS severity between sexes (*p* = 0.006). Male patients had a higher mean rank (105.17) than female patients (86.78), suggesting an association between male sex and greater disease severity. When demographic and functional parameters were compared between sexes, FVC (L), total lung volume, and FEV_1_ (L) were significantly higher in males. BMI was significantly higher in women (Table 5).

**Figure 3:**
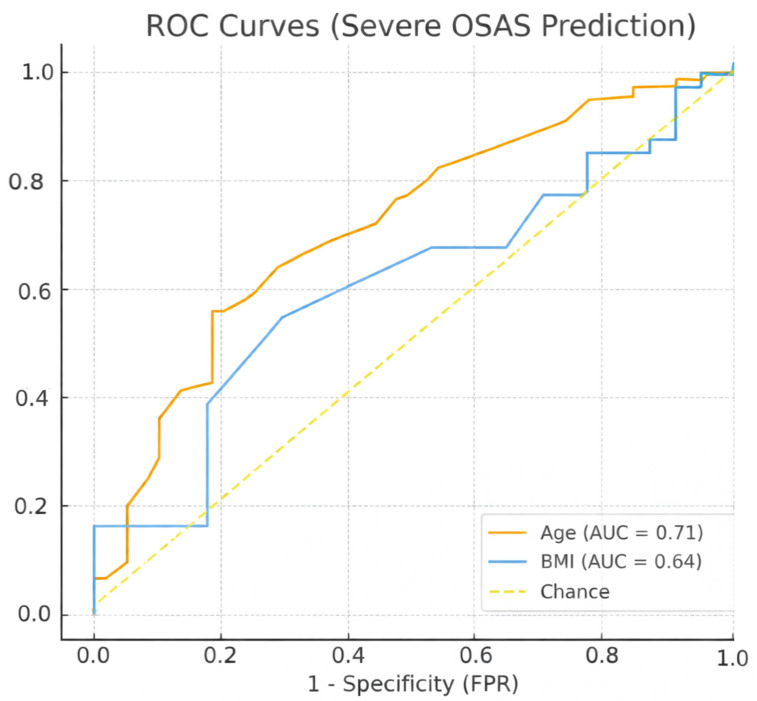
Receiver operating characteristic (ROC) curves for the prediction of severe obstructive sleep apnea syndrome (OSAS) according to age and body mass index (BMI). The area under the curve (AUC) values were 0.71 for age, 0.64 for BMI.

ROC analyses were performed for age and, BMIas predictors of severe OSAS (Figure 3). The strongest predictor was age (AUC = 0.71), with an optimal cut-off of 53.5 years (sensitivity 58.1%, specificity 76.3%). For BMI, the AUC was 0.64, with an optimal cut-off of 33.5 kg/m^2^ (sensitivity, 54.4%; specificity, 69.5%).

## 4. Discussion

Obstructive sleep apnea syndrome is a significant disorder characterized by recurrent upper airway obstruction or narrowing, resulting in intermittent hypoxia, sleep fragmentation, and frequent nocturnal awakenings [8]. Risk factors contributing to the development of OSAS include obesity, male sex, advanced age, craniofacial abnormalities, and ethnicity. In the elderly, several factors have been proposed to explain the increased prevalence of OSAS, including age-related upper airway narrowing, reduced activity of pharyngeal dilator muscles, changes in lung volumes, altered arousal thresholds, and instability of ventilatory control [1].

In this study, we examined the relationship between morphological lung volume and OSAS severity, a relationship that had not been previously investigated. Ordinal regression and General Linear Model analysis revealed that advancing age and higher BMI were associated with increased disease severity, while female sex acted as a protective factor. Neither CT-derived lung volume nor spirometric lung function was found to influence the severity of OSAS.

A meta-analysis reported BMI as the most commonly identified risk factor for OSAS, followed by age and sex, while hypertension, smoking, and alcohol consumption were less frequently reported [9]. In our study, BMI, age and sex were not only risk factors but also affected the degree of OSAS.

A study in pediatric patients with Down syndrome showed significantly higher BMI in those with OSAS [10]. Similarly, patients with overlap syndrome (OVS) were reported to have higher BMI compared with COPD patients, and BMI was positively correlated with AHI. Female sex was found to exert a protective effect in this subgroup as well [6]. Our results are consistent with these findings, with BMI being identified as a strong predictor of OSAS. The optimal BMI cut-off in our study was 34 kg/m^2^. While BMI is only an indirect marker of obesity, numerous studies have shown its strong association with OSAS severity. For example, individuals with BMI > 33 kg/m^2^ have been shown to exhibit higher oxygen desaturation and arousal indices, along with lower baseline oxygen saturation [11].

A study from Korea demonstrated that older age, higher body weight, and diabetes mellitus were independent risk factors for OSAS in patients with interstitial lung disease [12]. Similarly, male sex, higher BMI, and aging have been reported as independent predictors of severe OSAS in the elderly [13]. Our ROC analysis also identified age as the strongest predictor of severe OSAS (AUC = 0.71), with an optimal cut-off of 53.5 years. These findings are consistent with the existing literature, which highlights the positive association between age and OSAS severity.

Male sex increases the risk of OSAS, likely due to anatomical differences in upper airway structures, including total neck soft tissue volume and pharyngeal cross-sectional area [14,15,16]. The rise in OSAS prevalence and visceral fat mass after menopause suggests a protective role of female sex hormones [17,18,19]. In the same age group, men and obese individuals face greater risks compared with women, although menopause represents the most important predictor for women [20]. In our study, although women had higher BMI, the severity of OSAS was significantly higher in men. This result suggests that anatomical differences in the upper airway are more strongly associated with the severity of OSAS in men than BMI and lung volumes.

A meta-analysis showed that heavy smokers (>20 pack-years) had higher AHI and Epworth Sleepiness Scale scores and lower minimum oxygen saturation [21]. Another retrospective study found that AHI increased by 15.3% with each incremental rise in smoking exposure [22]. In our study, there was no significant relationship between AHI score and cigarette pack-years.

The ALEC study demonstrated reduced lung volumes in individuals at high risk for OSAS, as well as an independent association between higher OSAS symptom scores (snoring, apneas, and choking episodes) and faster declines in predicted FEV_1_ and FVC. Higher BMI was also independently associated with greater declines in predicted FVC [5]. These results align with our findings of negative correlations between BMI and lung volumes. However, despite this relationship, lung volume did not appear to directly influence OSAS severity in our cohort, likely due to the disease’s multifactorial nature. In ALEC, when analysis was restricted to individuals without BMI changes, only asthmatic patients showed significant associations between OSA symptoms and lung function decline [5]. Since our study excluded patients with asthma, this may explain the absence of such a relationship.

In pediatric OSAS, one study reported that FVC (% predicted) was lower in mild cases than in moderate and severe cases [4]. In contrast, our results demonstrated a negative correlation between FVC (% predicted) and AHI. This discrepancy may be due to differences in classification thresholds and the inclusion of children with possible comorbidities such as asthma. Other studies with BMI-matched controls have shown significant negative associations between OSAS severity and expiratory flows (e.g., FEF50, FEV_1_) [23], consistent with our findings.

Studies in obese adults with OSAS have shown significantly reduced pulmonary function compared with controls, with lower proximal (FEV_1_) and distal (MMEF, FEF25-75) flows, as well as higher levels of bronchial inflammation [24]. However, those studies did not exclude asthma or other conditions. In our study, by including only OSAS patients without comorbid lung disease, we observed no direct influence of lung function or volume on disease severity.

Our study showed that in the ordinal regression model, age demonstrated the greatest independent contribution to OSAS severity classification (Wald = 19.3), whereas BMI was the strongest predictor of raw AHI values in the GLM (ηp^2^ = 0.151). This discrepancy reflects the difference between categorizing disease severity and predicting the continuous magnitude of AHI. Indeed, BMI demonstrated the strongest correlation with OSAS severity, whereas morphological and functional lung volumes were negatively correlated with BMI. Although this suggests that lung volumes indirectly affect OSAS, they did not affect OSAS severity when BMI and other clinical factors were held constant. Similar to our study, Fabozzi et al. showed that there was no significant difference between OSAS severity and FEV_1_ (% predicted) and FVC (% predicted) [25]. The presence of multiple factors—including age, sex, race, obesity, comorbidities, genetic predisposition, OSAS phenotypes, environmental exposures, socioeconomic status, physical inactivity, and smoking—likely obscures any direct relationship between lung volume and OSAS severity [26]. It should also be noted that the classification of OSAS severity by AHI, first introduced in 1999 as a research tool, was not originally intended for clinical use. Recent studies have questioned its accuracy as a sole measure of disease severity [1]. This limitation may also explain why lung volume parameters did not correlate with AHI in our analysis. While severe OSAS is strongly associated with cardiovascular, neurocognitive, and metabolic disorders, such associations are less consistent and harder to establish in mild to moderate OSAS [26,27]. This inconsistency may have contributed to our negative findings.

The present study was limited to OSAS and did not include patients with CSAS. CSAS is a heterogeneous group of sleep-related breathing disorders characterized by the absence of intermittent respiratory effort during sleep. CSAS is caused by a disruption of neurological signals from the respiratory centers to the respiratory muscles, resulting in airflow stopping for at least 10 s. The main etiological causes are heart failure, opioid use, central neurological disorders and exposure to high altitude [28].

Our study has several limitations. These include its retrospective design, lack of data on upper airway anatomical details, absence of a matched control group, and the relatively small number of patients with mild and moderate OSAS. Larger, prospective studies with more homogeneous populations are needed to validate our findings. Moreover, due to the retrospective nature of our study, lung volume measurements may be affected by variations in patient inspiratory effort during imaging. Although standard breathing instructions were given to all patients, submaximal inspiratory effort cannot be entirely ruled out and remains a limitation of our volumetric analysis. Furthermore, patients with CSAS were not included in this study; therefore, the findings cannot be generalized to the broader sleep apnea population.

Importantly, this study is the first to assess CT-derived morphological lung volume in OSAS. Our results suggest that in patients without underlying lung disease, morphological lung volume behaves similarly to functional lung volume, as evidenced by strong correlations among total lung volume, FEV_1_, and FVC. Although OSAS severity was not directly related to lung volumes, reduced lung volume may still represent a risk factor for OSAS.

## 5. Conclusions

In this study, age and BMI were positively associated with OSAS severity, whereas female sex was inversely associated with disease severity. Functional and morphological lung volumes were positively correlated. Among all variables, age showed the strongest association with disease severity. OSAS severity increased in parallel with BMI. Moreover, BMI showed negative correlations with both morphological and functional lung volumes.

Although lung volumes were not directly associated with OSAS severity, our findings suggest that reduced lung volume may indirectly contribute to OSAS pathogenesis. Future prospective studies with larger and more homogeneous populations are needed to clarify these associations and further define the role of lung volume in OSAS.

## Figures and Tables

**Table 1:** Demographic and pulmonary function characteristics of the study population.

Variable	n	Minimum	Maximum	Mean ± SD
Age (years)	195	21	81	53.37 ± 11.78
Weight (kg)	195	51	150	94.87 ± 17.37
Height (cm)	195	140	193	167.06 ± 9.86
BMI (kg/m^2^)	195	20	58	34.19 ± 7.06
Smoking (pack-years)	48	3	80	31.04 ± 19.77
FVC (L)	195	0.65	6.78	3.45 ± 1.03
FVC (% predicted)	195	17	143	95.73 ± 18.38
FEV_1_ (L)	195	0.53	5.56	2.76 ± 0.83
FEV_1_ (% predicted)	195	15	148	93.50 ± 18.54
FEV_1_/FVC pre (%)	195	65.05	100	79.79 ± 5.29
Right lung volume (L)	166	0.99	4.61	2.46 ± 0.76
Left lung volume (L)	166	0.81	4.11	2.11 ± 0.72
Total lung volume (L)	166	1.72	8.72	4.56 ± 1.47

Note: Values are expressed as mean ± standard deviation (SD) unless otherwise indicated. Abbreviations: BMI: body mass index; FVC: forced vital capacity; FEV_1_: forced expiratory volume in one second.

**Table 2:** Demographic and pulmonary function characteristics according to sex.

Variable	Female (n = 76) Mean ± SD (Min–Max)	Male (n = 119) Mean ± SD (Min–Max)
Age (years)	53.64 ± 11.77 (24–80)	53.20 ± 11.83 (21–81)
Weight (kg)	92.64 ± 19.10 (51–144)	96.29 ± 16.10 (60–150)
Height (cm)	157.89 ± 6.10 (140–170)	172.90 ± 6.90 (155–193)
BMI (kg/m^2^)	37.26 ± 7.95 (20–58)	32.22 ± 5.64 (22–54)
Smoking (pack-years)	27.85 ± 20.30 (3–67)	32.35 ± 19.70 (6–80)
FVC (L)	2.69 ± 0.67 (1.30–4.15)	3.93 ± 0.93 (0.65–6.78)
FVC (% predicted)	99.64 ± 18.42 (59–143)	93.23 ± 17.99 (17–134)
FEV_1_ (L)	2.17 ± 0.57 (0.93–3.64)	3.12 ± 0.76 (0.53–5.56)
FEV_1_ (% predicted)	94.94 ± 19.45 (51–148)	92.58 ± 17.96 (15–140)
FEV_1_/FVC pre (%)	80.09 ± 4.96 (65.05–89.11)	79.59 ± 5.51 (70–100)
Right lung volume (L)	1.93 ± 0.48(0.99–3.05)	2.78 ± 0.71 (1.10–4.61)
Left lung volume (L)	1.57 ± 0.45 (0.81–2.61)	2.45 ± 0.65 (0.91–4.11)
Total lung volume (L)	3.49 ± 0.93 (1.72–5.66)	5.24 ± 1.34 (2.01–8.72)

Note: Values are expressed as mean ± standard deviation (SD) with minimum–maximum in parentheses. Abbreviations: BMI: body mass index; FVC: forced vital capacity; FEV_1_: forced expiratory volume in one second.

**Table 3:** Ordinal Logistic Regression Analysis of Factors Predictive of OSAS Severity.

Variables	Coefficient (β)	Std. Error	Wald	df	*p*-Value	95% Confidence Interval
Thresholds						
[OSAS Severity = 1.00]	4.294	1.765	5.916	1	0.015	0.834–7.753
[OSAS Severity = 2.00]	5.673	1.778	10.186	1	0.001	2.189–9.157
Predictors						
Age	0.071	0.016	19.259	1	<0.001	0.039–0.103
BMI	0.103	0.029	12.173	1	<0.001	0.045–0.160
Sex [Female]	−1.226	0.412	8.867	1	0.003	−2.033–−0.419
Smoking [User]	0.248	0.331	0.562	1	0.454	−0.401–0.898
FVC (L)	0.234	0.241	0.942	1	0.332	−0.239–0.706
Total Lung Volume	−0.163	0.153	1.137	1	0.286	−0.464–0.137

Notes: Statistical Model: Ordinal Logistic Regression was performed using the Complementary Log-log link function due to the asymmetrical distribution of the dependent variable (69.9% severe OSAS). Reference Categories: Male for sex and non-smoker for smoking status. Model Fit: The model significantly predicted the outcome (X^2^ = 43.432, *p* < 0.001) and met the assumption of proportional odds (Parallel Lines Test, *p* = 0.428). Abbreviations: BMI: Body Mass Index; df: Degrees of freedom; FVC: Forced Vital Capacity (pre-bronchodilator, liters); OSAS: Obstructive Sleep Apnea Syndrome.

**Table 4:** Univariate Analysis of Factors Predicting AHI Values (General Linear Model).

Source	Type III Sum of Squares	df	Mean Square	F	*p*-Value	Partial Eta Squared (ηp^2^)
Corrected Model	37,681.94	7	5383.14	9.67	<0.001	0.300
BMI	15,612.18	1	15,612.18	28.03	<0.001	0.151
Age	4460.39	1	4460.39	8.01	0.005	0.048
Sex	11,381.03	1	11,381.03	20.43	<0.001	0.115
Smoking Status	40.68	1	40.68	0.07	0.787	0.000
Total Lung Volume	28.60	1	28.60	0.05	0.821	0.000
FVC (L)	411.28	1	411.28	0.74	0.391	0.005
Sex × Smoking	5.15	1	5.15	0.01	0.923	0.000
Error	88,029.97	158	557.15			
Total	489,829.50	166				

Note: The General Linear Model was statistically significant (F = 9.67, *p* < 0.001) and accounted for 30% of the variance in AHI (R^2^ = 0.300). Levene’s test confirmed the equality of error variances (*p* = 0.948), indicating that the model assumptions were met. Abbreviations: AHI: Apnea–Hypopnea Index; BMI: Body Mass Index; FVC: Forced Vital Capacity; ηp^2^: Partial Eta Squared.

**Table 5:** Comparison of demographic and pulmonary function characteristics between female and male subjects.

Variable	Female (n = 76) Mean ± SD	Male (n = 119) Mean ± SD	*p*-Value
Age (years)	53.64 ± 11.78	53.20 ± 11.84	0.799
BMI (kg/m^2^)	37.26 ± 7.96	32.23 ± 5.64	<0.001
Smoking (pack-years)	27.86 ± 20.30 (n = 14)	32.35 ± 19.71 (n = 34)	0.480
FVC (L)	2.70 ± 0.67	3.94 ± 0.94	<0.001
FVC (% predicted)	99.64 ± 18.43	93.23 ± 17.99	0.017
FEV_1_ (L)	2.18 ± 0.57	3.13 ± 0.76	<0.001
FEV_1_ (% predicted)	94.95 ± 19.46	92.59 ± 17.96	0.388
FEV_1_/FVC (%)	80.09 ± 4.96	79.60 ± 5.52	0.525
Right lung volume (L)	1.93 ± 0.48 (n = 64)	2.78 ± 0.71 (n = 102)	<0.001
Left lung volume (L)	1.57 ± 0.45 (n = 64)	2.45 ± 0.65 (n = 102)	<0.001
Total lung volume (L)	3.49 ± 0.93 (n = 64)	5.24 ± 1.34 (n = 102)	<0.001

Note: Values are expressed as mean ± standard deviation (SD). Abbreviations: BMI: body mass index; FVC: forced vital capacity; FEV_1_: forced expiratory volume in one second.

## Data Availability

The datasets used and analysed during the current study are available from the corresponding author upon reasonable request.

## Abbreviations

The following abbreviations are used in this manuscript.

AHI	Apnea–Hypopnea Index
BMI	Body Mass Index
FEV_1_	Forced expiratory volume in one second.
FVC	Forced Vital Capacity
OSAS	Obstructive Sleep Apnea Syndrome
